# Supplementary material for: Long-Term Effects of Semaglutide and Sitagliptin on Circulating IGFBP-1, IGFBP-3 and IGFBP-rp1: Results from a One-Year Study in Type 2 Diabetes
Source: Int J Mol Sci. 2025 Oct 26;26(21):10404. doi: 10.3390/ijms262110404 (PMC12609315; doi:10.3390/ijms262110404)
Supplement: Supplementary file 1 [file ijms-26-10404-s001.zip › Supplementary Table S1.pdf]

Supplementary Table S1. Correlations between baseline main anthropometric, laboratory parameters and insulin like growth-factor 1 binding protein 1 (IGFBP-1) in patients with type 2 diabetes (T2DM) and controls

Baseline log_{10} IGFBP-1 vs.				
	Patients with T2DM		Controls	
	Correlation coefficient (r)	p-value	Correlation coefficient (r)	p-value
Age (year)	0.127	0.475	0.008	0.966
Body mass index (kg/m^2)	-0.012	0.950	-0.153	0.446
Waist circumference (cm)	-0.321	0.195	-0.351	0.119
Hemoglobin$_{A1c}$ (%)	0.181	0.314	-0.116	0.566
C-peptide (pmol/l)	-0.245	0.176	-0.149	0.556
Insulin (mU/l)	-0.318	0.076	-0.309	0.124
Creatinine (umol/l)	-0.205	0.252	-0.211	0.281
log_{10} hsCRP	0.285	0.120	0.494	0.037
log_{10} triglyceride	-0.106	0.556	-0.246	0.206
Cholesterol (mmol/l)	0.061	0.734	0.110	0.580
non-HDLC (mmol/l)	0.040	0.827	0.341	0.065
LDL-C (mmol/l)	0.105	0.568	0.200	0.308
Apolipoprotein B100 (g/l)	-0.074	0.697	0.361	0.305
HDL-C (mmol/l)	0.316	0.078	-0.079	0.688
Apolipoprotein AI (g/l)	0.351	0.057	0.331	0.079
sVCAM-1 (ng/ml)	0.490	0.003	-0.015	0.937
sICAM-1 (ng/ml)	0.057	0.752	0.012	0.949

Abbreviations: hsCRP, high sensitive C-reactive protein; HDL-C, high-density lipoprotein cholesterol; LDL-C, low-density lipoprotein cholesterol; sICAM-1, soluble intercellular adhesion molecule-1; sVCAM-1, soluble vascular cell adhesion molecule-1.

Supplementary Table S2. Correlations between baseline main anthropometric, laboratory parameters and insulin like growth-factor 1 binding protein 3 (IGFBP-3) in patients with type 2 diabetes (T2DM) and controls

Baseline IGFBP-3 vs.				
	Patients with T2DM		Controls	
	Correlation coefficient (r)	p-value	Correlation coefficient (r)	p-value
Age (year)	-0.027	0.879	0.301	0.107
Body mass index (kg/m^2)	-0.035	0.856	0.050	0.809
Waist circumference (cm)	-0.370	0.131	0.186	0.421
$Hemoglobin_{A1c}$ (%)	0.003	0.989	-0.156	0.437
C-peptide (pmol/l)	-0.063	0.731	0.048	0.851
Insulin (mU/l)	-0.054	0.771	-0.030	0.884
Creatinine (umol/l)	-0.202	0.259	-0.015	0.942
log_{10} hsCRP	0.270	0.141	0.303	0.222
log_{10} triglyceride	0.172	0.340	-0.112	0.570
Cholesterol (mmol/l)	0.521	0.002	-0.069	0.725
non-HDLC (mmol/l)	0.500	0.004	0.205	0.277
LDL-C (mmol/l)	0.565	<0.001	0.009	0.960
Apolipoprotein B100 (g/l)	0.509	0.004	0.346	0.328
HDL-C (mmol/l)	0.208	0.254	0.062	0.754
Apolipoprotein AI (g/l)	0.240	0.201	0.084	0.667
sVCAM-1 (ng/ml)	-0.096	0.579	0.132	0.496
sICAM-1 (ng/ml)	0.261	0.142	0.307	0.105

Abbreviations: hsCRP, high sensitive C-reactive protein; HDL-C, high-density lipoprotein cholesterol; LDL-C, low-density lipoprotein cholesterol; sICAM-1, soluble intercellular adhesion molecule-1; sVCAM-1, soluble vascular cell adhesion molecule-1.

Supplementary Table S3. Correlations between baseline main anthropometric, laboratory parameters and insulin like growth-factor 1 binding protein related protein 1 (IGFBP-rp1) in patients with type 2 diabetes (T2DM) and controls

Baseline IGFBP-rp1 vs.				
	Patients with T2DM		Controls	
	Correlation coefficient (r)	p-value	Correlation coefficient (r)	p-value
Age (year)	-0.014	0.938	0.192	0.310
Body mass index (kg/m^2)	0.166	0.364	0.204	0.307
Waist circumference (cm)	0.193	0.443	-0.154	0.506
Hemoglobin$_{A1c}$ (%)	0.128	0.479	-0.034	0.867
C-peptide (pmol/l)	0.400	0.043	0.444	0.065
Insulin (mU/l)	0.259	0.159	0.254	0.211
Creatinine (umol/l)	0.288	0.110	-0.086	0.665
log_{10} hsCRP	-0.014	0.940	0.136	0.590
log_{10} triglyceride	0.073	0.692	0.287	0.139
Cholesterol (mmol/l)	0.260	0.151	-0.131	0.510
non-HDLC (mmol/l)	0.218	0.239	0.141	0.458
LDL-C (mmol/l)	0.141	0.450	-0.085	0.666
Apolipoprotein B100 (g/l)	0.146	0.449	-0.235	0.513
HDL-C (mmol/l)	-0.194	0.295	-0.323	0.094
Apolipoprotein AI (g/l)	-0.156	0.418	0.254	0.184
sVCAM-1 (ng/ml)	0.262	0.141	0.376	0.044
sICAM-1 (ng/ml)	-0.025	0.889	0.176	0.362

Abbreviations: hsCRP, high sensitive C-reactive protein; HDL-C, high-density lipoprotein cholesterol; LDL-C, low-density lipoprotein cholesterol; sICAM-1, soluble intercellular adhesion molecule-1; sVCAM-1, soluble vascular cell adhesion molecule-1.
