# Supplementary figures and images for: Long-Term Effects of Semaglutide and Sitagliptin on Circulating IGFBP-1, IGFBP-3 and IGFBP-rp1: Results from a One-Year Study in Type 2 Diabetes
Source: Int J Mol Sci. 2025 Oct 26;26(21):10404. doi: 10.3390/ijms262110404 (PMC12609315; doi:10.3390/ijms262110404)

**Supplementary Figure S1. Study design flowchart**

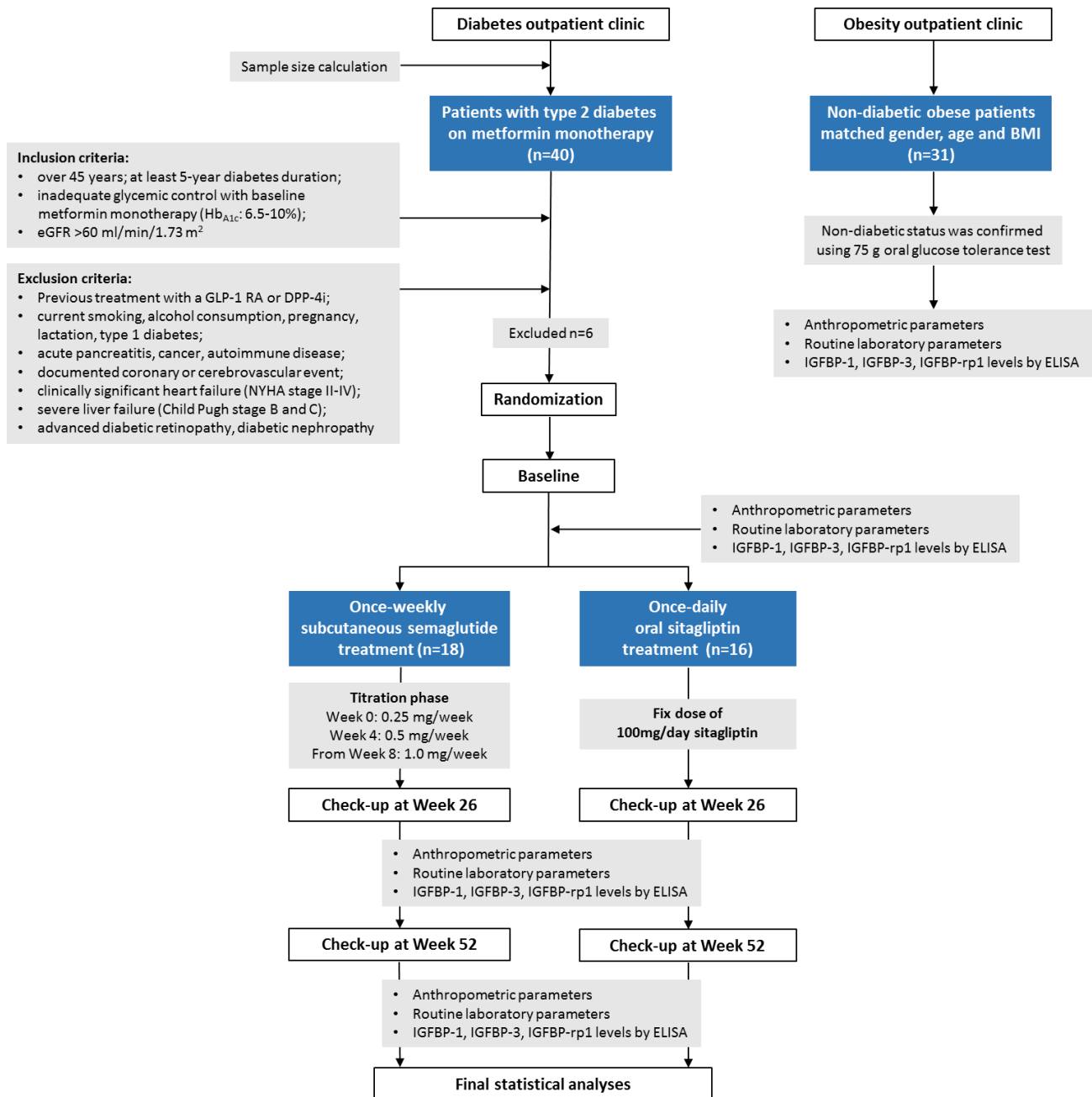

Supplement: Supplementary file 1 [file ijms-26-10404-s001.zip › Supplementary Figure S1 r1.pdf]
